# Differential brainstem connectivity according to sex and menopausal status in healthy male and female individuals

**DOI:** 10.1186/s13293-025-00709-4

**Published:** 2025-04-18

**Authors:** Lisa A. Kilpatrick, Arpana Church, David Meriwether, Swapna Mahurkar-Joshi, Vince W. Li, Jessica Sohn, Juliana Reist, Jennifer S. Labus, Tien Dong, Jonathan P. Jacobs, Bruce D. Naliboff, Lin Chang, Emeran A. Mayer

**Affiliations:** 1 Vatche and Tamar Manoukian Division of Digestive Diseases, Department of Medicine, David Geffen School of Medicine, University of California Los Angeles, Los Angeles, CA USA; 2 G. Oppenheimer Center for Neurobiology of Stress and Resilience, David Geffen School of Medicine, University of California Los Angeles, Los Angeles, CA USA; 3 Goodman-Luskin Microbiome Center, University of California Los Angeles, Los Angeles, CA USA; 4 Division of Gastroenterology, Hepatology and Parenteral Nutrition, VA Greater Los Angeles Healthcare System, Los Angeles, CA USA; 5 Brain Research Institute, Gonda (Goldschmied) Neuroscience and Genetics Research Center, University of California Los Angeles, Los Angeles, CA USA

**Keywords:** Sex differences, Estrogen, Neuroimaging, Brainstem nuclei

## Abstract

**Background:**

Brainstem nuclei play a critical role in both ascending monoaminergic modulation of cortical function and arousal, and in descending bulbospinal pain modulation. Even though sex-related differences in the function of both systems have been reported in animal models, a complete understanding of sex differences, as well as menopausal effects, in brainstem connectivity in humans is lacking. This study evaluated resting-state connectivity of the dorsal raphe nucleus, right and left locus coeruleus complex (LCC), and periaqueductal gray (PAG) according to sex and menopausal status in healthy individuals. In addition, relationships between systemic estrogen levels and brainstem-network connectivity were examined in a subset of participants.

**Methods:**

Resting-state fMRI was performed in 47 healthy male (age, 31.2 ± 8.0 years), 53 healthy premenopausal female (age, 24.7 ± 7.3 years; 22 in the follicular phase, 31 in the luteal phase), and 20 postmenopausal female participants (age, 54.6 ± 7.2 years). Permutation Analysis of Linear Models (5000 permutations) was used to evaluate differences in brainstem-network connectivity according to sex and menopausal status, controlling for age. In 10 males and 17 females (9 premenopausal; 8 postmenopausal), estrogen and estrogen metabolite levels in plasma and stool were determined by liquid chromatography-mass spectrometry/mass spectrometry. Relationships between estrogen levels and brainstem-network connectivity were evaluated by partial least squares analysis.

**Results:**

Left LCC-executive control network connectivity showed an overall sex difference (p = 0.02), with higher connectivity in females than in males; however, this was mainly due to differences between males and premenopausal females (p = 0.008). Additional sex differences were dependent on menopausal status: PAG-default mode network (DMN) connectivity was higher in postmenopausal females than in males (p = 0.04), and PAG-sensorimotor network (SMN) connectivity was higher in premenopausal females than in males (p = 0.03) and postmenopausal females (p = 0.007). Notably, higher free 2-hydroxyestrone levels in stool were reliably associated with higher PAG-SMN and PAG-DMN connectivity in premenopausal females (p < 0.01).

**Conclusions:**

Healthy females show higher brainstem-network connectivity involved in cognitive control, sensorimotor function, and self-relevant processes than males, dependent on their menopausal status. Further, 2-hydroxyestrone, implicated in pain, may modulate PAG connectivity in premenopausal females. These findings may relate to differential vulnerabilities to chronic stress-sensitive disorders at different life stages.

**Supplementary Information:**

The online version contains supplementary material available at 10.1186/s13293-025-00709-4.

## Abstract (plain language summary)

Males and females show differences in the likelihood to develop chronic pain disorders, as well as anxiety and depression, which often accompany chronic pain, across the lifespan. Brainstem nuclei are small structures in the brain yet show powerful influences across the entire brain. Here, we investigated differences in the connectivity of several brainstem nuclei with major brain networks involved in stress responsiveness and pain modulation between healthy males and healthy premenopausal and postmenopausal females, as well as their association with estrogen levels in the body. We found higher connectivity between the locus coeruleus and the executive control network in females, especially premenopausal females, than in males, which may relate to differences in preferred information processing strategies and anxiety symptoms. We also found higher connectivity between the periaqueductal gray and the default mode network in postmenopausal females and the sensorimotor network in premenopausal females than in males. Further, higher levels of 2-hydroxyestrone, an estrogen metabolite that plays a role in visceral pain sensitivity, was related to periaqueductal gray connectivity in premenopausal females. These latter findings may relate to differences in vulnerability to visceral pain disorders at different life stages. These results help in identifying potential contributors to sex differences in vulnerability to chronic disorders that reduce the quality of life, before a disorder develops.

**Supplementary Information:**

The online version contains supplementary material available at 10.1186/s13293-025-00709-4.

## Highlights

- We evaluated differences in the connectivity of several brainstem nuclei with major brain networks between healthy premenopausal and postmenopausal females and males, as well as relationships between connectivity and estrogen levels in plasma and stool.
- Premenopausal females showed (1) higher connectivity between the left locus coeruleus complex and executive control network compared to that in males, and (2) higher connectivity between the periaqueductal gray and sensorimotor network compared to that in males and postmenopausal females.
- Postmenopausal females showed higher connectivity between the periaqueductal gray and default mode network compared to that in males.
- Levels of free 2-hydroxyestrone, an estrogen metabolite implicated in visceral pain, in plasma and stool were associated with periaqueductal gray connectivity in premenopausal females.
- These results may relate to differential vulnerabilities to chronic pain and stress-sensitive disorders across the lifespan.

**Supplementary Information:**

The online version contains supplementary material available at 10.1186/s13293-025-00709-4.

## Background

Brainstem nuclei, including the locus coeruleus complex (LCC), dorsal raphe nucleus (DRN), and periaqueductal gray (PAG), play critical roles in ascending monoaminergic modulation of brain and vital functions, as well as in endogenous descending pain modulation. Alterations in these modulations have been demonstrated in chronic pain disorders, as well as in anxiety and depression, which are often comorbid with each other [1–5]. The LCC is the primary source of noradrenergic innervation of the forebrain and exerts a powerful modulatory role over cognitive and affective functions via widespread cortical and subcortical projections [6]. The DRN is the primary serotonergic nucleus in the central nervous system and modulates several vital functions, including mood, appetite, and sleep, through ascending projections to many cortical and subcortical brain regions [7]. The PAG is involved in integrated descending modulation of pain, as well as in autonomic and behavioral responses to threat. It has reciprocal connections with prefrontal and emotion-regulation regions and receives top-down input from the orbitofrontal cortex and insula [4].

Sex differences in LCC, DRN, and PAG structure and function exist [8, 9]. For instance, animal studies suggest that the LCC arousal system is more sensitive to corticotropin-releasing factor, which is involved in stress, in females than in males [10, 11]. In addition, neuroimaging studies suggest that the LCC has stronger connectivity with the hippocampus in males than in females [12], consistent with animal research demonstrating greater LCC noradrenergic input to the hippocampus in males than in females [13]. Animal research indicates PAG/DRN involvement in sex differences in pain-related behaviors, promoting anti-nociception in males and pain-related locomotor behaviors in females [9]. Females have greater risk for numerous chronic pain disorders, as well as greater risk for anxiety and depression [14]. Sex differences in the connectivity of brainstem nuclei may be related to differential vulnerability to chronic pain conditions and comorbid mood disorders.

Estrogens are known to impact LCC, DRN, and PAG function. Estrogens modulate LCC output, generally increasing noradrenergic levels in target regions [15]. Estrogens also increase the expression of tryptophan hydroxylase, a rate-limiting enzyme for serotonergic synthesis, in the DRN, reducing anxiety and increasing active coping behavior in animal studies [16–18]. The PAG contains a large population of estrogen receptors, contributing to known sex differences in response to morphine administration, with a greater antinociceptive effect in males than in females [19].

Menopause is associated with increased risk for anxiety and depression [20], as well as for some chronic pain conditions, such as fibromyalgia, migraine, and back pain [21]. However, the role of estrogen in the risk for and severity of symptoms in disorders of gut brain interaction (DGBI) in premenopausal and postmenopausal females is incompletely understood. We previously found that postmenopausal females with irritable bowel syndrome (IBS) have greater overall IBS symptom severity and worse health-related quality of life [22]. However, in a large global epidemiology study in individuals with IBS and other DGBI, premenopausal females reported a greater frequency of gastrointestinal symptoms compared to males and postmenopausal females [23]. In addition, another study found that menopause may be associated with less risk for DGBI such as IBS [24]. Changes in brainstem connectivity during menopause may contribute to these changes in symptom severity in postmenopausal females. However, a complete understanding of sex and menopausal effects on brainstem connectivity, especially in humans, is lacking.

In the current study, we aimed to evaluate sex and menopausal status effects on resting-state connectivity of the DRN, left and right LCC, and PAG with major brain networks involved in stress responsiveness, pain modulation, and emotion regulation, including the central autonomic network (CAN), default mode network (DMN), emotional arousal network (EAN), executive control network (ECN), salience network (SAL), and sensorimotor network (SMN), in healthy individuals, as well as relationships between affected connectivity and anxiety and somatic symptoms. In addition, relationships between estrogen levels and brainstem-network connectivity were examined in a subset of participants.

## Methods

### Participants

Healthy male and female participants were recruited from the Los Angeles area through advertisements and local clinics. Exclusion criteria were as follows: chronic pain disorder, major neurological condition or vascular disease, current or past psychiatric disorder, substance use disorder, use of centrally acting medications, pregnant or breastfeeding, weight > 400 lbs, and MRI contraindications (e.g. metal implants). In addition, individuals with < 8 min of low-motion resting-state fMRI data (with low motion defined as framewise displacement < 0.2 mm) were excluded.

All participants underwent a medical history and physical examination. Participants also underwent the Mini-International Neuropsychiatric Interview to assess past/current psychiatric disorders [25]. Sex was self-reported as the sex assigned at birth; prior to October 2023, male and female were the only options presented to the participants, after which additional options including intersex and ‘none of these’ were presented. For consistency, only those indicating male or female at birth were included. Menopausal status was determined by the following criteria: females who had regular menses within the previous 12 months were premenopausal; females who did not have menses within the previous 12 months, with decreased serum estradiol (E2) and increased serum follicle-stimulating hormone levels based on normal laboratory values were considered postmenopausal, in accordance with STRAW + 10 criteria [26]. Among premenopausal female participants, menstrual cycle phase was determined using urine ovulation kits. Pregnancy or childbirth within the past 12 months were exclusion criteria for all female participants.

This study was approved by the Institutional Review Board at the University of California, Los Angeles’s Office of Protection for Research Subjects (Nos. 20–000540 and 20–000515). All participants provided written informed consent.

### Questionnaires

Participants completed questionnaires on anxiety and somatic symptoms to enable the examination of the behavioral correlates of implicated brainstem connectivity. These included the Hospital Anxiety and Depression (HAD) scale, as a measure of current anxiety symptoms (total score: 0–21) [27]; State-Trait Anxiety Inventory (STAI), as a measure of trait anxiety (total score: 20–80) [28]; Perceived Stress Scale (PSS), as a measure of ongoing stress burden (total score: 0–40) [29]; and the Patient Health Questionnaire-15 (PHQ-15; total score: 0–15) [30] and Pennebaker Inventory of Limbic Languidness (PILL; total score: 0–54) as measures of the bothersomeness and frequency of common somatic symptoms, respectively [31].

### Imaging acquisition and preprocessing

Participants underwent neuroimaging on a 3.0 T Siemens Prisma MRI scanner (Siemens, Erlangen, Germany). Structural MRI (T1-weighted and T2-weighted) and resting-state fMRI scans were performed in accordance with Human Connectome Project (HCP) protocols (version 4.3), with a field of view optimized for the brainstem. Specifically, acquisition parameters for high-resolution T1-weighted scans were as follows: echo time, 1.81 ms; repetition time, 2500 ms; slice thickness, 0.8 mm; number of slices, 208; voxel matrix, 320 × 300; and voxel size, 1.0 × 1.0 × 0.8 mm. Parameters for the T2-weighted scans were as follows: echo time, 564 ms; repetition time, 3200 ms; slice thickness, 0.8 mm; number of slices, 208; voxel matrix, 320 × 300; and voxel size, 1.0 × 1.0 × 0.8 mm. Resting-state scans were obtained in anterior–posterior and posterior-anterior directions (8 min each; total duration, 16 min), with eyes open. Parameters for the resting-state scan were as follows: echo time, 37 ms; repetition time, 1000 ms; flip angle, 52 deg; slice thickness, 2 mm; number of slices, 88; voxel matrix, 104 × 104; and voxel size, 2.0 × 2.0 × 2.0 mm. Spin echo fieldmaps were also acquired in anterior–posterior and posterior-anterior directions for distortion correction.

Images were preprocessed using the ABCD-HCP pipeline (https://github.com/DCAN-Labs/abcd-hcp-pipeline) [32], which is based on the HCP pipeline [33]. Briefly, structural images underwent bias field correction, volume segmentation and cortical surface reconstruction using FreeSurfer 6.0 [34], and MNI registration; resting-state images underwent distortion and bias field correction, realignment, MNI registration via T1-weighted registration, and intensity normalization. Additionally, resting-state images were denoised by regressing out head motion parameters and white matter and cerebrospinal fluid signals, which were refined using respiratory motion filtering (13.2–18.6 breaths per minute), and band-pass filtering (0.008, 0.09 Hz) [35].

Denoised resting-state images were parcellated using the Destrieux atlas for cortical regions [36], Harvard–Oxford atlas for subcortical regions, and ascending arousal network atlas for brainstem regions [37]. Fisher-transformed connectivity matrices were created using 8 min of low-movement data (defined as framewise displacement < 0.20 mm). The correlation between each brainstem region of interest (DRN, left LCC, right LCC, and PAG) and each network of interest (CAN, DMN, EAN, ECN, SAL, and SMN) was calculated as the average of the pairwise correlations between the brainstem region and all of regions belonging to the network. Regions included in each network of interest are indicated in Table 1.

**Table 1:** Network definitions based on the Destrieux cortical and Harvard–Oxford subcortical atlases

Network	Brain regions	Atlas labels
Central Autonomic Network (CAN)	Orbitofrontal cortex, anterior cingulate cortex, anterior insula, amygdala, midbrain nuclei	Transverse frontopolar gyri and sulci, middle-anterior part of the cingulate gyrus and sulcus, short insular gyri, orbital gyri, gyrus rectus, subcallosal gyrus, anterior segment of the circular sulcus of the insula, lateral orbital sulcus, medial orbital sulcus, orbital sulci, amygdala, ventral diencephalon
Default Mode Network (DMN)	Medial prefrontal cortex, posterior cingulate cortex, inferior parietal cortex, lateral temporal cortex	Transverse frontopolar gyri and sulci, posterior-dorsal part of the cingulate gyrus, posterior-ventral part of the cingulate gyrus, angular gyrus, supramarginal gyrus, precuneus, lateral aspect of the superior temporal gyrus, planum temporale
Emotional Arousal Network (EAN)	Medial and ventrolateral prefrontal cortex, anterior cingulate cortex, parahippocampal gyrus, hippocampus, amygdala	Transverse frontopolar gyri and sulci, anterior part of the cingulate gyrus and sulcus, opercular part of the inferior frontal gyrus, orbital part of the inferior frontal gyrus, triangular part of the inferior frontal gyrus, parahippocampal gyrus, gyrus rectus, subcallosal gyrus, suborbital sulcus, hippocampus, amygdala
Executive Control Network (ECN)	Dorsolateral prefrontal cortex, superior parietal cortex	Middle frontal gyrus, superior parietal lobule, inferior frontal sulcus, middle frontal sulcus, intraparietal sulcus and transverse parietal sulci, subparietal sulcus
Salience Network (SAL)	Orbitofrontal cortex, anterior mid-cingulate cortex, anterior insula	Transverse frontopolar gyri and sulci, middle-anterior part of the cingulate gyrus and sulcus, short insular gyri, orbital gyri, gyrus rectus, anterior segment of the circular sulcus of the insula, lateral orbital sulcus, medial orbital sulcus, orbital sulci
Sensorimotor Network (SMN)	Primary/secondary somatosensory cortices, primary/supplementary motor cortices, posterior mid-cingulate cortex, posterior insula, thalamus, basal ganglia	Paracentral lobule and sulcus, subcentral gyrus and sulci, middle-posterior part of the cingulate gyrus and sulcus, superior frontal gyrus, long insular gyrus and central sulcus of the insula, postcentral gyrus, precentral gyrus, central sulcus, inferior segment of the circular sulcus of the insula, superior segment of the circular sulcus of the insula, superior frontal sulcus, postcentral sulcus, inferior part of the precentral sulcus, superior part of the precentral sulcus, thalamus, caudate, putamen, pallidum, nucleus accumbens

### Determination of free and total estrogens in plasma and stool

In a subset of participants (10 males; 9 premenopausal females, with 4 and 5 in follicular and luteal phases, respectively, during scanning and sample collection; and 8 postmenopausal females), the free and total (total = free + conjugated) levels of 15 estrogens and estrogen metabolites in plasma and stool were determined by liquid chromatography-mass spectrometry/mass spectrometry (LC–MS/MS). Estrogen species analyzed included the three main types of estrogen, estrone (E1), estradiol (E2), estriol (E3), as well as 12 additional estrogen metabolites: 2-hydroxyestrone (2OHE1), 2-methoxyestrone (2MeOE1), 2-hydroxyestradiol (2OHE2), 2-methoxyestradiol (2MeOE2), 2-hydroxyestrone-3-methyl ether (3MeOE1), 4-hydroxyestrone (4OHE1), 4-methoxyestrone (4MeOE1), 4-methoxyestradiol (4MeOE2), 16α-hydroxyestrone (16aOHE1), 17-epiestriol (17epiE3), 16-ketoestradiol (16ketoE2), and 16-epiestriol (16epiE3). Both sample preparation and LC–MS/MS methods followed previously published methods [38–40], with minor modifications.

#### Plasma sample preparation

Plasma was prepared with and without enzymatic hydrolysis. For the determination of free estrogen, 100 uL plasma was combined with stable heavy isotope internal standards and 300 uL of basic reaction buffer consisting of 0.15 M acetate buffer pH 4.5 containing 1.0 mg/mL L-ascorbic acid. For the determination of total estrogen, 100 uL plasma was prepared as above, but 15 uL of β-glucuronidase/aryl-sulfatase (from Helix pomatia) was also added and samples were kept gently rocking at 37 °C overnight. For both free and total samples, isopropanol was added (5% v/v) and lipids were extracted with 400 cc supported liquid extraction cartridges (Biotage LLC) using 3 × 1.5 mL dichloromethane extractions and dried under argon.

#### Stool sample preparation

Stool for both free and total estrogen (100 mg each) was combined with internal standards and 1 mL basic reaction buffer and subjected to bead mill homogenization (Biotage Lysera). Stool for total estrogen was combined with 15 uL deconjugation enzyme and reacted overnight as above. Both sample sets were combined with 500 ul acetonitrile; re-homogenized; and centrifuged. Supernatant was loaded into 2 cc supported liquid extraction cartridges (Biotage) and extracted with 3 × 2 mL DCM followed by drying under argon.

#### Sample derivatization

Dried plasma and stool samples were combined with 100 uL of 1 mg/mL-acetone dansyl chloride and 100 uL 0.1 M sodium bicarbonate buffer pH 9.2; reacted for 20 min at 60 °C; centrifuged; and transferred to LCMS vials.

#### LC–MS/MS

LC–MS/MS analysis was performed on an Agilent 1290/SCIEX QTrap 5500 system using principles of tuning, method validation, quality control, calibration, and quantitation that we previously described [41]. MS/MS settings for derivatized estrogens and stable heavy isotope internal standards were determined using reference calibration and internal standards (Steraloids); values were comparable to those previously reported [38, 39]. Liquid chromatography was performed using a Kinetex C18 1.7 um 2.1 × 150 mm column (Phenomenex), while the gradient transitioned from 90% water/0.1% formic acid to 95% acetonitrile/0.1% formic acid across 20 min. Concentration of all estrogens was determined as ng/mL-plasma or ng/5 mg-stool.

### Statistical analysis

Group differences in characteristics were evaluated by analyses of variance, with the exception of PHQ-15 and PILL scores. Because of strongly skewed distributions, PHQ-15 and PILL scores were dichotomized by a median split, such that no/minimal somatic burden was defined as a score of 0 or 1 on the PHQ-15/PILL and more than minimal somatic burden was defined as a score of 2 or more PHQ-15/PILL. Group differences in the frequency of more than minimal somatic burden were evaluated by chi-squared analysis.

Permutation analysis of linear models with non-parametric combination (NPC) was used to evaluate brainstem-network connectivity according to sex and menopausal status, with 5000 permutations [42, 43]. NPC combines the test statistics of separate analyses into a single joint statistic, the significance of which is assessed through synchronized permutations for each of the separate tests [43, 44]. In this case, the test statistics of separate analyses of males vs females in the follicular phase, males vs. females in the luteal phase and males vs postmenopausal females, controlling for age, were combined into a joint statistic representing male vs female participants across menstrual status/phase categories (i.e., an overall sex difference) and joint statistics representing differences according to menopausal status (i.e., premenopausal females vs males, premenopausal females vs postmenopausal females, and postmenopausal females vs males). Familywise error (FWE)-corrected p-values < 0.05 were considered significant.

Partial least squares correlation (PLSC) analysis was applied to examine relationships between estrogen/behavioral data and brainstem-network connectivity with significant sex/menopausal status effects, using plscmd in Matlab (http://www.rotman-baycrest.on.ca/pls) [45]. PLSC analysis is a multivariate analytical technique that identifies weighted patterns of variables in two blocks of variables that maximally covary with each other (i.e. latent variables) and is appropriate for data with multicollinearity. As metabolite pathways are interrelated, multicollinearity in the estrogen data was expected. In the present study, Block 1 comprised brainstem-network data and Block 2 comprised estrogen data (free and total levels in plasma and stool) or behavioral data (HAD anxiety, STAI trait anxiety, PSS, PHQ-15, and PILL). Permutation testing (5000 permutations) was used to assess latent variable significance and bootstrap estimation was applied (5000 samples) to assess the reliability of individual saliences within the latent variable. In the present study, we report latent variables with any statistically reliable saliences according to the bootstrap ratio (BSR). For behavioral analyses, BSRs of magnitude 1.96 or greater (corresponding to p < 0.05) were considered statistically reliable. For estrogen analyses, given the limited sample size, a more stringent cutoff was adopted; BSRs of magnitude 2.58 or greater (corresponding to p < 0.01) were considered statistically reliable.

## Results

### Participant characteristics

From the 50 male and 75 female participants (21 postmenopausal), 3 males and 2 females (1 postmenopausal) were excluded from analysis due to insufficient low-motion resting-state data. Accordingly, 47 healthy male participants (mean age: 31.2 ± 8.0 years), 53 healthy premenopausal female participants (mean age: 24.7 ± 7.3 years), and 20 healthy postmenopausal female participants (mean age: 54.6 ± 7.2 years) were included in the analysis. Among the premenopausal female participants, 22 were scanned during the follicular phase and 31 were scanned during the luteal phase of their menstrual cycle. None of the postmenopausal female participants were taking hormone replacement therapy at the time of scanning. In addition, there were no cases of induced menopause (e.g. no cases of bilateral oophorectomy). Although HAD anxiety scores were low in this healthy population, analysis of variance revealed significant differences in HAD anxiety (F(2,117) = 4.09, p = 0.02), with significantly higher scores in premenopausal female participants than in male participants (p = 0.005). In addition, there was a significant difference in the frequency of more than minimal PHQ-15 scores, with higher frequency in female participants than in male participants (χ^2^ = 6.7, p = 0.03) (Table 2).

**Table 2:** Participant characteristics

	N	Age (yr)	PSS	HAD Anxiety	STAI Trait Anxiety	PHQ-15 > 2	PILL > 2
Males	47	31.2 ± 8.0	11.7 ± 5.8	3.0 ± 2.1	47.8 ± 9.9	21 (45%)	23 (48%)
Premenopausal females	53	24.7 ± 7.3	12.0 ± 5.7	4.5 ± 2.9	48.2 ± 9.9	36 (68%)	37 (70%)
Postmenopausal females	20	54.6 ± 7.2	9.5 ± 4.4	3.4 ± 2.9	46.3 ± 9.1	14 (70%)	10 (50%)
			F (2,117) = 1.5, p = 0.23	F (2,117) = 4.09, p = 0.02	F (2,117) = 0.3, p = 0.77	χ^2^ = 6.7, p = 0.03	χ^2^ = 5.1, p = 0.07

Data are shown as mean ± standard deviation or number (%)

*HAD* Hospital Anxiety and Depression, *PSS* Perceived Stress Scale, *STAI* State-Trait Anxiety Inventory, *PHQ-15* Patient Health Questionnaire-15), *PILL* Pennebaker Inventory of Limbic Languidness

### Sex and menopausal status effects on brainstem connectivity

**Fig. 1:**
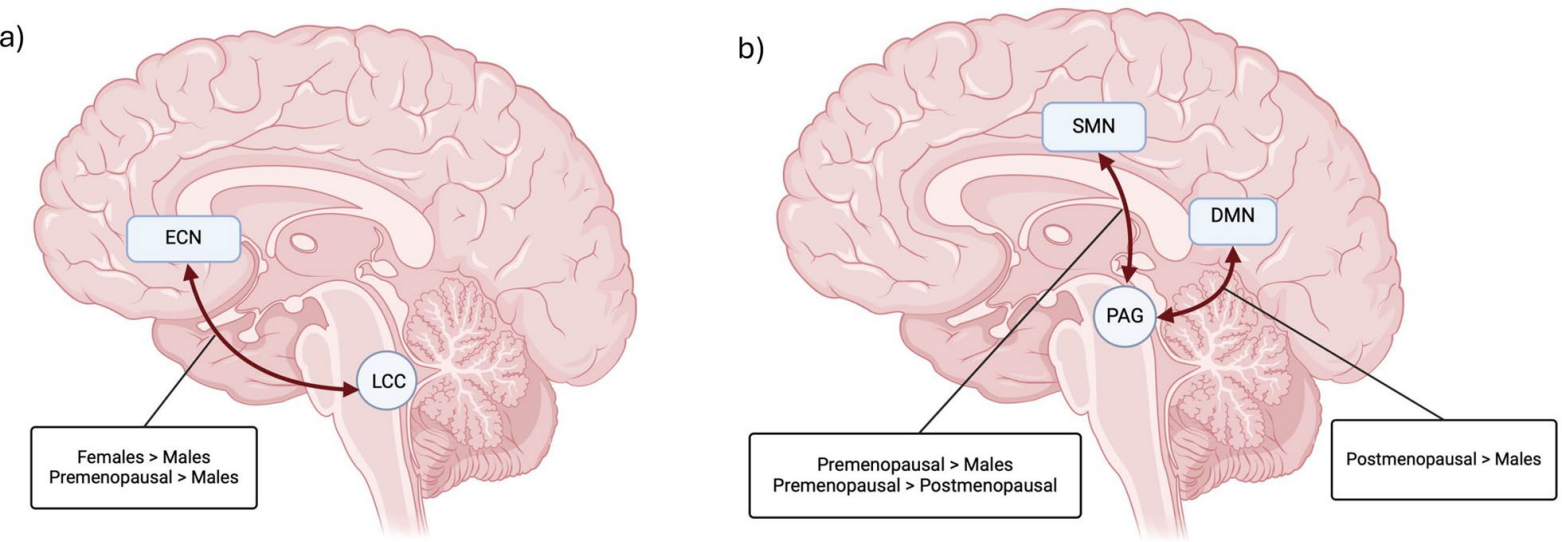
Sex and menopausal status effects on brainstem connectivity. **a** Brainstem-network connectivity showing a significant overall sex difference (all females vs males). Left LCC connectivity with the ECN was higher in female participants than in male participants (p_fwe_ = 0.02); however, a subgroup analysis indicated that this difference was mainly driven by premenopausal female participants (p_fwe_ = 0.008). **b** Brainstem-network connectivity showing a significant difference between premenopausal and postmenopausal female participants and/or a sex difference dependent on menopausal status. PAG connectivity with the SMN was higher in premenopausal female participants than in male (p_fwe_ = 0.03) and postmenopausal female participants (p_fwe_ = 0.007). In contrast, PAG connectivity with the DMN was significantly higher in postmenopausal female participants than in male participants (p_fwe_ = 0.03). This simplified figure does not show the distributed and somewhat overlapping nature of the regions in the networks (which are listed in Table 1). *DMN* default mode network, *ECN* executive control network, *p*_*fwe*_ familywise error-corrected p-value, *LCC* locus coeruleus complex, *PAG* periaqueductal gray, *SMN* sensorimotor network

Significant differences in brainstem-network connectivity according to sex and menopausal status are summarized in Fig. 1.

NPC analysis revealed a significant overall sex difference in left LCC-ECN connectivity (p_fwe_ = 0.02), with higher connectivity in all female participants than in male participants. However, subgroup analysis according to menopausal status revealed that the overall sex difference was mainly due to significantly higher connectivity in premenopausal female participants than in male participants (p_fwe_ = 0.008). No significant difference was observed between postmenopausal female and male participants (p_fwe_ = 0.69). There was a trend toward higher connectivity in premenopausal than in postmenopausal female participants (p_fwe_ = 0.051).

NPC analysis revealed significant sex differences in PAG connectivity dependent on menopausal status. Specifically, PAG-DMN connectivity was significantly higher in postmenopausal female participants compared to male participants, a difference not seen between premenopausal female and male participants (p_fwe_ = 0.03; p_fwe_ = 0.35, respectively). Additionally, PAG-SMN connectivity was significantly higher in premenopausal female participants than in male (p_fwe_ = 0.03) and postmenopausal female participants (p_fwe_ = 0.007).

### Relationships between estrogens and brainstem connectivity

As indicated above, left LCC-ECN, PAG-DMN, and PAG-SMN connectivity showed significant group differences and were, thus, submitted to PLSC analysis in a subset of participants with estrogen data to evaluate relationships between connectivity and estrogen levels within each group. Specifically, estrogen and connectivity data for the three connections were simultaneously submitted to PLSC analysis in each participant group (i.e. males, premenopausal females, postmenopausal females). A summary of estrogen levels is provided in Additional file 1; as expected, levels were lower, with a more restricted range, in male and postmenopausal female participants compared to that in premenopausal female participants.

**Fig. 2:**
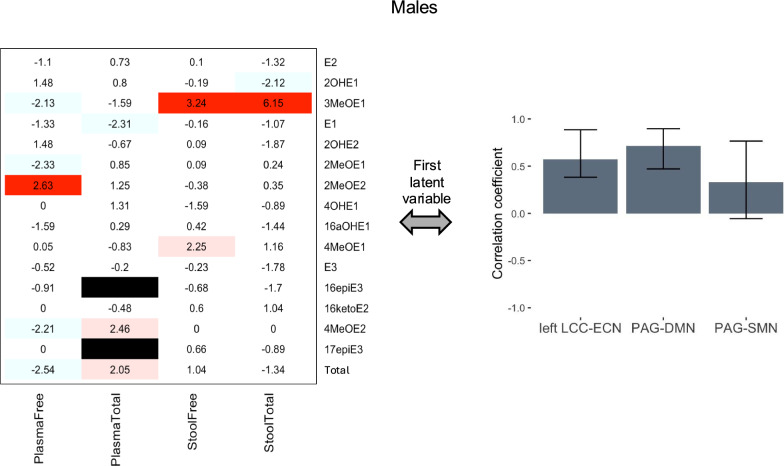
Relationships between estrogen levels and connectivity in males. In male participants, the first latent variable reflected estrogen levels reliably associated with left LCC-ECN and PAG-DMN connectivity; no other associations were statistically reliable. Heatmaps indicate the BSR of each evaluated estrogen and estrogen metabolite for the connectivity pattern shown in the corresponding bar graph, with statistically reliable positive ratios (at p < 0.01, i.e. BSR > 2.58) highlighted in red. Additional estrogens and estrogen metabolites with a BSR meeting a lower threshold of p < 0.05 are shown in light blue (BSR < − 1.96) or pink (BSR > 1.96). The plasma total levels of 16epiE3 and 17epiE3 were removed from analysis due to false positives (black bars). The bars in the bar graphs indicate the correlation between each connection and the estrogen pattern shown in the corresponding heatmap and the whiskers indicate the 95% confidence interval (confidence intervals entirely above or below zero were considered to indicate significance). This simplified figure does not show the distributed and somewhat overlapping nature of the regions in the networks (which are listed in Table 1). *BSR* bootstrap ratio, *DMN* default mode network, *ECN* executive control network, *LCC* locus coeruleus complex, *PAG* periaqueductal gray, *SMN* sensorimotor network, *E1* estrone, *E2* estradiol, *E3* estriol: *2OHE1* 2-hydroxyestrone, *2MeOE1* 2-methoxyestrone, *2OHE2* 2-hydroxyestradiol, *2MeOE2* 2-methoxyestradiol, *3MeOE1* 2-hydroxyestrone-3-methyl ether, *4OHE1* 4-hydroxyestrone, *4MeOE1* 4-methoxyestrone, *4MeOE2* 4-methoxyestradiol, *16aOHE1* 16α-hydroxyestrone, *17epiE3* 17-epiestriol, *16ketoE2* 16-ketoestradiol, *16epiE3* 16-epiestriol, Total, summation of all free or total (free + conjugated) estrogens and estrogen metabolites, dependent on category

The first latent variable in the PLSC analysis of connectivity-estrogen relationships in male participants accounted for 47.3% of the cross-block variance (p = 0.05) and revealed that higher levels of free 2MeOE2 in plasma, and free and total 3MeOE1 in stool, were reliably associated with higher left LCC-ECN and PAG-DMN connectivity on bootstrap testing (Fig. 2). No other latent variables had statistically reliable saliences on bootstrap testing.

**Fig. 3:**
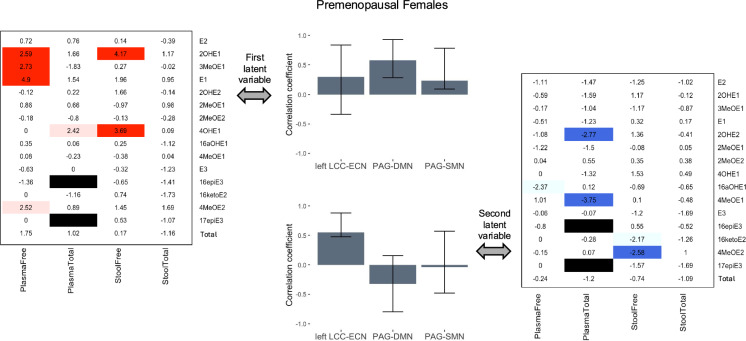
Relationships between estrogen levels and connectivity in premenopausal females. In premenopausal female participants, the first latent variable reflected estrogen levels reliably associated with PAG-SMN and PAG-DMN connectivity, while the second variable reflected estrogen levels reliably associated with left LCC-ECN connectivity. Heatmaps indicate the BSR of each evaluated estrogen and estrogen metabolite for the connectivity pattern shown in the corresponding bar graph, with statistically reliable ratios (at p < 0.01) highlighted in dark blue (BSR < − 2.58) or red (BSR > 2.58). Additional estrogens and estrogen metabolites with a BSR meeting a lower threshold of p < 0.05 are shown in light blue (BSR < − 1.96) or pink (BSR > 1.96). The plasma total levels of 16epiE3 and 17epiE3 were removed from analysis due to false positives (black bars). The bars in the bar graphs indicate the correlation between each connection and the estrogen pattern shown in the corresponding heatmap and the whiskers indicate the 95% confidence interval (confidence intervals entirely above or below zero were considered to indicate significance). Note: This simplified figure does not show the distributed and somewhat overlapping nature of the regions in the networks (which are listed in Table 1). *BSR* bootstrap ratio, *DMN* default mode network, *ECN* executive control network, *LCC* locus coeruleus complex, *PAG* periaqueductal gray, *SMN* sensorimotor network, *E1* estrone, *E2* estradiol, *E3* estriol: *2OHE1* 2-hydroxyestrone, *2MeOE1* 2-methoxyestrone, *2OHE2* 2-hydroxyestradiol, *2MeOE2* 2-methoxyestradiol, *3MeOE1* 2-hydroxyestrone-3-methyl ether, *4OHE1* 4-hydroxyestrone, *4MeOE1* 4-methoxyestrone, *4MeOE2* 4-methoxyestradiol, *16aOHE1* 16α-hydroxyestrone, *17epiE3* 17-epiestriol, *16ketoE2* 16-ketoestradiol, *16epiE3* 16-epiestriol; Total, summation of all free or total (free + conjugated) estrogens and estrogen metabolites, dependent on category

The first latent variable in the PLSC analysis of connectivity-estrogen relationships in premenopausal female participants accounted for 43.9% of the cross-block variance (p = 0.49) and revealed that higher levels of free E1 and 2OHE1 in plasma and free 2OHE1 and 4OHE1 in stool, were reliably associated with higher PAG-DMN and PAG-SMN connectivity on bootstrap testing. The second latent variable accounted for 36.8% of the cross-block variance, (p = 0.47) and revealed that lower levels of total 2OHE2 and 4MeOE1 in plasma, and lower levels of free 4MeOE2 in stool, were reliably associated with higher left LCC-ECN connectivity on bootstrap testing (Fig. 3).

**Fig. 4:**
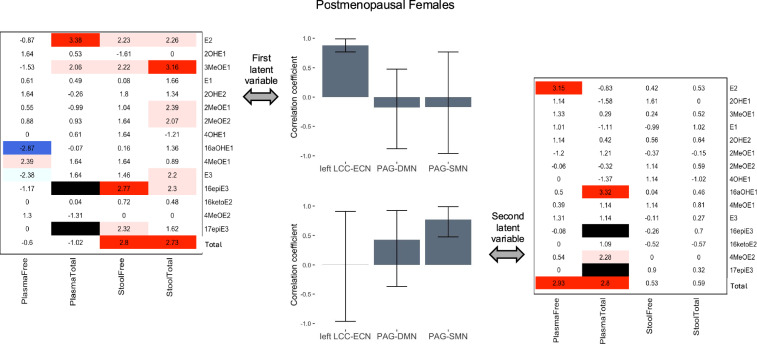
Relationships between estrogen levels and connectivity in postmenopausal females. In postmenopausal female participants, the first latent variable reflected estrogen levels reliably associated with left LCC-ECN connectivity, while the second variable reflected estrogen levels reliably associated with PAG-SMN connectivity. Heatmaps indicate the BSR of each evaluated estrogen and estrogen metabolite for the connectivity pattern shown in the corresponding bar graph, with statistically reliable ratios (at p < 0.01) highlighted in dark blue (BSR < − 2.58) or red (BSR > 2.58). Additional estrogens and estrogen metabolites with a BSR meeting a lower threshold of p < 0.05 are shown in light blue (BSR < − 1.96) or pink (BSR > 1.96). The plasma total levels of 16epiE3 and 17epiE3 were removed from analysis due to false positives (black bars). The bars in the bar graphs indicate the correlation between each connection and the estrogen pattern shown in the corresponding heatmap and the whiskers indicate the 95% confidence interval (confidence intervals entirely above or below zero were considered to indicate significance). Note: This simplified figure does not show the distributed and somewhat overlapping nature of the regions in the networks (which are listed in Table 1). *BSR* bootstrap ratio, *DMN* default mode network, *ECN* executive control network, *LCC* locus coeruleus complex, *PAG* periaqueductal gray, *SMN* sensorimotor network, *E1* estrone, *E2* estradiol, *E3* estriol: *2OHE1* 2-hydroxyestrone, *2MeOE1* 2-methoxyestrone, *2OHE2* 2-hydroxyestradiol, *2MeOE2* 2-methoxyestradiol, *3MeOE1* 2-hydroxyestrone-3-methyl ether, *4OHE1* 4-hydroxyestrone, *4MeOE1* 4-methoxyestrone, *4MeOE2* 4-methoxyestradiol, *16aOHE1* 16α-hydroxyestrone, *17epiE3* 17-epiestriol, *16ketoE2* 16-ketoestradiol, *16epiE3* 16-epiestriol; Total, summation of all free or total (free + conjugated) estrogens and estrogen metabolites, dependent on category

The first latent variable in the PLSC analysis of connectivity-estrogen relationships in postmenopausal female participants accounted for 43.8% of the cross-block variance (p = 0.05) and revealed that higher levels of total E2 and lower levels of free 16aOHE1 in plasma, and higher levels of free 16epiE3, total 3MeOE1, and total-free and total-total estrogens (summation all free estrogens and estrogen metabolites and summation of all free + conjugated estrogens and estrogen metabolites, respectively) in stool, were reliably associated with higher left LCC-ECN connectivity on bootstrap testing. The second latent variable accounted for 30.2% of the cross-block variance (p = 0.21) and revealed that higher levels of free E2, total 16aOHE1, and total-free and total-total estrogens in plasma were reliably associated with higher PAG-SMN connectivity on bootstrap testing (Fig. 4).

### Relationships between anxiety and somatic symptoms and brainstem connectivity

Relationships between left LCC-ECN, PAG-DMN, and PAG-SMN connectivity and anxiety and somatic symptoms were similarly examined in the total sample and in each participant group (i.e. males, premenopausal females, postmenopausal females).

The first latent variable in the PLSC analysis of relationships between brainstem connectivity and anxiety/somatic symptoms in the total sample accounted for 44.4% of the cross-block variance (p = 0.10) and revealed that higher HAD anxiety (BSR = 1.97, p = 0.049) was reliably associated higher left LCC-ECN connectivity on bootstrap testing (r = 0.10, 95% confidence interval [95% CI] 0.02–0.26). No other latent variables had statistically reliable saliences on bootstrap testing.

The first latent variable in the PLSC analysis of relationships between brainstem connectivity and anxiety and somatic symptoms in male participants accounted for 65.8%of the cross-block variance (p = 0.002) and revealed that higher perceived stress (BSR = 2.78, p = 0.005) and trait anxiety (BSR = 4.05, p < 0.001) were reliably associated with higher PAG-DMN connectivity on bootstrap testing (r = 0.40, 95% CI 0.21–0.59). No other latent variables had statistically reliable saliences on bootstrap testing.

The first latent variable in the PLSC analysis of relationships between brainstem connectivity and anxiety and somatic symptoms in premenopausal female participants accounted for 54.5% of the cross-block variance (p = 0.06) and revealed that higher PHQ-15 scores (BSR = 2.29, p = 0.02) were reliably associated with higher left LCC-ECN connectivity on bootstrap testing (r = 0.21, 95% CI 0.08–0.44). In addition, the second latent variable accounted for 36.1% of the cross-block variance (p = 0.36) and revealed that higher PILL scores (BSR = 2.40, p = 0.02) were reliably associated with higher PAG-SMN connectivity on bootstrap testing (r = 0.28, 95% CI 0.02–0.46).

PLSC analysis of relationships between brainstem connectivity and anxiety and somatic symptoms in postmenopausal female participants did not reveal any statistically reliable associations.

## Discussion

The present study evaluated differences in resting-state connectivity between specific brainstem nuclei (DRN, left and right LCC, and PAG) and key brain networks (CAN, DMN, EAN, ECN, SAL, and SMN) implicated in stress responsiveness and pain modulation according to sex and menopausal status in healthy individuals. We found a significant overall sex difference in left LCC-ECN connectivity, with generally higher connectivity in female participants than in male participants, mainly driven by higher connectivity in premenopausal female participants. There was also significantly higher PAG-SMN connectivity in premenopausal female participants than in male participants and in premenopausal female participants than in postmenopausal female participants. In contrast, significantly higher PAG-DMN connectivity was present in postmenopausal female participants than in male participants. Further, relationships between left LCC-ECN, PAG-SMN, and PAG-DMN and estrogen levels in plasma and stool, as well as anxiety and somatic symptoms, were observed.

### Sex differences in LCC resting-state connectivity may relate to known differences in information processing priorities

The left LCC showed higher connectivity with the ECN in female participants, especially premenopausal females, than in male participants. The ECN comprises lateral prefrontal and parietal regions and supports executive functions such as working memory, selective attention, and cognitive control [46]. Animal and human studies suggest that enhanced functional coupling of the LCC with the ECN is associated with increased goal-directed attention and decreased impulsivity [47, 48].

LCC noradrenergic signaling biases perception, attention, and memory toward more salient stimuli by selectively amplifying the activity of priority mechanisms operating at the moment [49]. Substantial evidence suggests that while males show a greater preference for allocentric knowledge (e.g., describing objects/others independent of one’s own perspective), with a greater reliance on hippocampal-based strategies [8], females show a greater preference for egocentric knowledge (e.g., describing objects in terms of one’s own spatial perspective and using more privileged information in inference), with a greater reliance on working memory processes mediated by frontal regions, such as those in the ECN [8, 50]. We speculate that these previously reported sex-related biases in information processing priorities are operating under resting-state condition and underlie the observed difference in left LCC connectivity between males and females. Further, anxiety and egocentricism are related, with greater reliance on egocentric perspective-taking/mentalizing in those experiencing anxiety [51]. Thus, under our speculation, the observed greater LCC-ECN connectivity in premenopausal females than in males may be related to the higher current anxiety symptom scores in premenopausal female participants than in male participants. Consistent with this, higher HAD anxiety scores were associated with left LCC-ECN connectivity in the total sample, with a trend (p = 0.08) towards association among premenopausal female participants. In addition, left LCC-ECN connectivity was associated with higher PHQ-15 in premenopausal female participants. Although the PHQ-15 is not specifically focused on anxiety, it includes some items on somatic symptoms of anxiety (e.g. heart racing, shortness of breath, nausea, dizziness, stomachaches) and had shared variance with HAD anxiety in the present sample of premenopausal females (r = 0.48).

Interestingly, unlike the left LCC, the right LCC did not show any sex differences in connectivity. A recent mixed-sex study of LCC connectivity gradients found greater relationships between age, anxiety/depression symptoms, and cognitive performance for the left LCC than for the right LCC [52]. Additional research suggests that neurodegenerative disorders affect the left LCC more than the right LCC [53, 54]. Thus, the left LCC may be more pliable or sensitive than the right LCC. However, further research is needed.

In premenopausal female participants, decreased levels of mainly metabolites in the 2-hydroxylation pathway of estrogen metabolites, including plasma 2OHE2, were associated with higher left LCC-ECN connectivity. Metabolites of the 2-hydroxylation pathway are generally considered to have weak estrogenic activity; however, 2OHE2 has structural similarities to catecholamines and can compete with noradrenaline in the brain [55]. Thus, in premenopausal female participants, circulating 2OHE2 may interact with LC noradrenergic output, modulating left LCC-ECN connectivity. In contrast, in male participants, higher levels of methylated estrogen metabolites, including 2MeOE2 in plasma, and in postmenopausal female participants, higher levels of total E2 in plasma, were associated with higher left LCC-ECN connectivity (i.e., connectivity more similar to that of premenopausal female participants). 2MeOE2 was one of the more abundant estrogen metabolites in plasma in the male participants in the present study and E2 is a major endogenous estrogen. These results suggest that estrogens may affect LCC-ECN connectivity in males and postmenopausal females, but the effects may be more apparent for metabolites with relatively higher levels, as levels were generally lower in these individuals.

### Sex differences in PAG-SMN resting-state connectivity may relate to known sex differences in pain processing

The PAG showed higher connectivity with the SMN in premenopausal female participants than in male and postmenopausal female participants. The present results are consistent with a previous neuroimaging study that reported greater PAG connectivity with sensorimotor-related brain regions in healthy females than in healthy males [56], and add to the literature by showing a dependence on menopausal status.

The SMN comprises sensorimotor, mid-cingulate and superior frontal cortices, as well as the posterior insula, thalamus, and basal ganglia [57, 58]. The SMN is involved in central processing and modulation of visceral and somatic sensory information and both the PAG and SMN are involved in pain processing. Previous studies in patient populations indicate that increased connectivity of the PAG with the SMN, or specific regions within the SMN, may be associated with an increased risk of the development of chronic pain following mild traumatic brain injury [59] and increased central sensitization symptoms in patients with fibromyalgia [60]. In the present study, we found that higher PAG-SMN connectivity was associated with higher PILL scores, suggesting a relationship between PAG-SMN connectivity and increased somatic symptom burden, in premenopausal female participants. Numerous studies suggest that somatic symptom burden contributes to an increased risk for the development of chronic pain conditions [61, 62]. Thus, our findings align with an increased vulnerability to chronic pain conditions in premenopausal females than in males [23, 63]. However, a mixed-sex study in healthy individuals reported that resting-state connectivity between the PAG and SMN positively correlated with conditioned pain modulation, suggesting more efficient endogenous pain modulation with increased connectivity [64]. Primary somatosensory cortex modulates sensory gain and nociception, with outputs originating from layer 5 of the cortex and projecting to subcortical targets, including the PAG, comprising an anti-nociceptive pathway, and outputs originating from layer 6 of the cortex and projecting to the thalamus, which is also a component of the SMN and interacts with the PAG, comprising a pro-nociceptive pathway [65]. Thus, the interpretation of increased PAG-SMN connectivity is complicated and may require a finer-grained analysis.

However, one notable finding in the analysis of connectivity-estrogen relationships was that, in premenopausal female participants, increased plasma and stool free 2OHE1 was associated with increased PAG-SMN connectivity. 2OHE1 has been shown to increase nociceptor activation via transient receptor potential ankyrin 1 (TRPA1) and transient receptor potential vanilloid type-1 (TRPV1) channels in a mouse model of uterine pain [66]. Peripheral TRPA1 and TRPV1 sensitization have been implicated in visceral pain disorders, including DGBI [67–69]. However, central factors may be necessary for the persistence of visceral hypersensitivity [70]. TRPV1 receptors are expressed in the PAG and antagonists applied to the dorsolateral PAG can reduce anxiety-like and nociceptive behavior in animal models [71, 72]. Thus, estrogen metabolites may modulate the increased PAG-SMN connectivity in premenopausal females, potentially heightening somatic symptom burden and risk for chronic pain disorders such as DGBI.

### Sex differences in PAG-DMN resting-state connectivity may relate to known sex differences in risk for posttraumatic stress disorder and stress-induced analgesia

In the present study, PAG-DMN connectivity was significantly lower in male participants than in postmenopausal female participants; however, there was a positive association between PAG-DMN connectivity and trait anxiety (assessed by the STAI) and perceived stress (assessed by the PSS) in male participants, suggesting that, in males, acute and chronic feelings of worry and distress is associated with connectivity similar to that of premenopausal female participants. The DMN comprises the medial prefrontal cortex, posterior cingulate cortex, precuneus, inferior parietal cortices, and lateral temporal cortices, and is involved in self-referential processes [73, 74]. Additionally, building upon animal research, human brain imaging studies implicate DMN regions in threat processing, with the posterior cingulate cortex and medial prefrontal cortex involved in evaluating threat cues and modulating responses to threat, respectively [75]. A mixed-sex neuroimaging study reported increased PAG-DMN connectivity two weeks after a car accident as predictive of the development of posttraumatic stress disorder within 6 months [76]. In female patients with temporomandibular disorder, higher connectivity between PAG and posterior cingulate and medial prefrontal cortices is associated with pain rumination [77]. Thus, we speculate that increased PAG-DMN connectivity in postmenopausal females, and males with higher trait anxiety, may be associated with maladaptive coping strategies that contribute to risk for posttraumatic stress disorder [78]. Generally, females have higher risk for posttraumatic stress disorder than males [79]. Some studies suggest that risk for posttraumatic stress disorder in females peaks around menopause [80], which may explain why the sex difference in PAG-DMN connectivity was more robust for postmenopausal female participants than for premenopausal female participants.

### Limitations

The present study has several limitations. The number of postmenopausal female participants was relatively small, limiting the power to detect differences between postmenopausal and premenopausal females and sex differences that emerge or reverse after menopause. Additionally, although we controlled for age, bias may exist in comparisons between males and postmenopausal females because of limited overlap in age; thus, future studies with a greater representation of older males is needed to confirm the robustness of our results. Further, in the PLSC analyses, follicular and luteal females were combined into a single group due to the limited sample size, resulting in heterogeneity that may have contributed to non-significance on permutation testing. Therefore, we focused on salience reliability in our reporting. A larger sample, allowing follicular and luteal females to represented in separate groups, may result in improved power to detect significant latent variables that more precisely explain the variance. Additionally, although sex was treated as binary in the present analysis, sex is not strictly binary, as variation exists (e.g., in chromosomes, reproductive organs, hormones) that defies binary classification. Other limitations arise from the small size of brainstem nuclei, which can be difficult to delineate. We used a brainstem atlas based mostly on postmortem data to delineate the LCC, DRN, and PAG; however, imprecision in nuclei boundaries may have affected the connectivity estimates. Additionally, we investigated the overall connectivity of the LCC, DRN, and PAG, without consideration of differential connectivity within each of these brainstem regions. Although small, these regions show variations in connectivity, with a rostral-caudal connectivity gradient in the LC and subregions in the DRN and PAG with differential connectivity supporting various functions [81]. This may have contributed to the lack of significant sex differences in DRN connectivity. However, a finer-grained analysis is beyond the scope of the present study. Additionally, as a major limitation of correlational studies, the causality or directionality of interactions could not be addressed. Finally, the present study was exploratory in nature and estrogen data were available in a limited subset of participants; thus, the results should be interpreted with caution and further research is required to rigorously confirm the present findings.

## Conclusions

The present study expands the limited research on sex and menopausal effects on brainstem connectivity, and their relationships with various estrogens, in humans. We found that healthy females show higher left LCC and PAG connectivity with networks involved in cognitive control, and sensorimotor function and self-relevant processes, respectively, than males, dependent on their menopausal status. Although such differences may show benefits under optimal conditions, they may also relate to differential vulnerabilities to chronic pain and stress-sensitive disorders at different life stages. In particular, PAG connectivity with the SMN may be modulated by circulating 2OHE1 and associated with somatic symptoms in premenopausal females. Given the known role of 2OHE1 in peripheral and central sensitization processes, we speculate that this contributes to an increased risk for chronic pain disorders such as DGBI in premenopausal females. However, future studies are needed in patients with chronic pain.

## Supplementary Information

Supplementary Material 1. Mean estrogen levels, with standard errors, in males, premenopausal females, and postmenopausal females. Plasma data is provided in units of ng/mL-plasma, while stool data is provided in units of ng/5 mL-stool. E1, estrone; E2, estradiol; E3, estriol: 2OHE1, 2-hydroxyestrone; 2MeOE1, 2-methoxyestrone; 2OHE2, 2-hydroxyestradiol; 2MeOE2, 2-methoxyestradiol; 3MeOE1, 2-hydroxyestrone-3-methyl ether; 4OHE1, 4-hydroxyestrone; 4MeOE1, 4-methoxyestrone; 4MeOE2, 4-methoxyestradiol; 16aOHE1, 16α-hydroxyestrone; 17epiE3, 17-epiestriol; 16ketoE2, 16-ketoestradiol; 16epiE3, 16-epiestriol; Total, summation of all free or totalestrogens and estrogen metabolites, dependent on category.

## Data Availability

Data are available from the corresponding authors on reasonable request.

## References

[1] Tache Y, Martinez V, Million M, Rivier J. Corticotropin-releasing factor and the brain-gut motor response to stress. Can J Gastroenterol. 1999;13:18A-25A. 10.1155/1999/375916. 10202204 10.1155/1999/375916

[2] Hale MW, Shekhar A, Lowry CA. Stress-related serotonergic systems: implications for symptomatology of anxiety and affective disorders. Cell Mol Neurobiol. 2012;32(5):695–708. 10.1007/s10571-012-9827-1. 22484834 10.1007/s10571-012-9827-1 PMC3378822

[3] Mayer EA, Tillisch K. The brain-gut axis in abdominal pain syndromes. Annu Rev Med. 2011;62:381–96. 10.1146/annurev-med-012309-103958. 21090962 10.1146/annurev-med-012309-103958 PMC3817711

[4] George DT, Ameli R, Koob GF. Periaqueductal gray sheds light on dark areas of psychopathology. Trends Neurosci. 2019;42(5):349–60. 10.1016/j.tins.2019.03.004. 30955857 10.1016/j.tins.2019.03.004

[5] Peng WH, Kan HW, Ho YC. Periaqueductal gray is required for controlling chronic stress-induced depression-like behavior. Biochem Biophys Res Commun. 2022;593:28–34. 10.1016/j.bbrc.2022.01.025. 35051779 10.1016/j.bbrc.2022.01.025

[6] Mouton PR, Pakkenberg B, Gundersen HJ, Price DL. Absolute number and size of pigmented locus coeruleus neurons in young and aged individuals. J Chem Neuroanat. 1994;7(3):185–90. 10.1016/0891-0618(94)90028-0. 7848573 10.1016/0891-0618(94)90028-0

[7] Michelsen KA, Schmitz C, Steinbusch HW. The dorsal raphe nucleus–from silver stainings to a role in depression. Brain Res Rev. 2007;55(2):329–42. 10.1016/j.brainresrev.2007.01.002. 17316819 10.1016/j.brainresrev.2007.01.002

[8] Ycaza Herrera A, Wang J, Mather M. The gist and details of sex differences in cognition and the brain: how parallels in sex differences across domains are shaped by the locus coeruleus and catecholamine systems. Prog Neurobiol. 2019;176:120–33. 10.1016/j.pneurobio.2018.05.005. 29772255 10.1016/j.pneurobio.2018.05.005 PMC6485927

[9] Yu W, Pati D, Pina MM, Schmidt KT, Boyt KM, Hunker AC, et al. Periaqueductal gray/dorsal raphe dopamine neurons contribute to sex differences in pain-related behaviors. Neuron. 2021;109(8):1365–80. 10.1016/j.neuron.2021.03.001. 33740416 10.1016/j.neuron.2021.03.001 PMC9990825

[10] Bangasser DA, Curtis A, Reyes BA, Bethea TT, Parastatidis I, Ischiropoulos H, et al. Sex differences in corticotropin-releasing factor receptor signaling and trafficking: potential role in female vulnerability to stress-related psychopathology. Mol Psychiatry. 2010;15(9):96–904. 10.1038/mp.2010.66. 10.1038/mp.2010.66 PMC2935505 20548297

[11] Bangasser DA, Eck SR, Ordones SE. Sex differences in stress reactivity in arousal and attention systems. Neuropsychopharmacology. 2019;44(1):129–39. 10.1038/s41386-018-0137-2. 30022063 10.1038/s41386-018-0137-2 PMC6235989

[12] Zhang S, Hu S, Chao HH, Li CS. Resting-state functional connectivity of the locus coeruleus in humans: in comparison with the ventral tegmental area/substantia Nigra pars compacta and the effects of age. Cereb Cortex. 2016;26(8):3413–27. 10.1093/cercor/bhv172. 26223261 10.1093/cercor/bhv172 PMC4961017

[13] Sun P, Wang J, Zhang M, Duan X, Wei Y, Xu F, et al. Sex-related differential whole-brain input atlas of locus coeruleus noradrenaline neurons. Front Neural Circuits. 2020;14:53. 10.3389/fncir.2020.00053. 33071759 10.3389/fncir.2020.00053 PMC7541090

[14] Osborne NR, Davis KD. Sex and gender differences in pain. Int Rev Neurobiol. 2022;164:277–307. 10.1016/bs.irn.2022.06.013. 36038207 10.1016/bs.irn.2022.06.013

[15] Bangasser DA, Wiersielis KR, Khantsis S. Sex differences in the locus coeruleus-norepinephrine system and its regulation by stress. Brain Res. 2016;1641:177–88. 10.1016/j.brainres.2015.11.021. 26607253 10.1016/j.brainres.2015.11.021 PMC4875880

[16] McEwen B. Estrogen actions throughout the brain. Recent Prog Horm Res. 2002;57:357–84. 10.1210/rp.57.1.357. 12017552 10.1210/rp.57.1.357

[17] Donner N, Handa RJ. Estrogen receptor beta regulates the expression of tryptophan-hydroxylase 2 mRNA within serotonergic neurons of the rat dorsal raphe nuclei. Neuroscience. 2009;163(2):705–18. 10.1016/j.neuroscience.2009.06.046. 19559077 10.1016/j.neuroscience.2009.06.046 PMC2740745

[18] Campos GV, de Noronha SR, de Souza AA, Lima PM, Abreu AR, Chianca-Jr D, de Menezes RC. Estrogen receptor beta activation within dorsal raphe nucleus reverses anxiety-like behavior induced by food restriction in female rats. Behav Brain Res. 2019;357–358:57–64. 10.1016/j.bbr.2018.03.029. 29567265 10.1016/j.bbr.2018.03.029

[19] Averitt DL, Eidson LN, Doyle HH, Murphy AZ. Neuronal and glial factors contributing to sex differences in opioid modulation of pain. Neuropsychopharmacology. 2019;44(1):155–65. 10.1038/s41386-018-0127-4. 29973654 10.1038/s41386-018-0127-4 PMC6235988

[20] Alblooshi S, Taylor M, Gill N. Does menopause elevate the risk for developing depression and anxiety? Results from a systematic review. Australas Psychiatry. 2023;31(2):165–73. 10.1177/10398562231165439. 36961547 10.1177/10398562231165439 PMC10088347

[21] Gibson CJ, Li Y, Bertenthal D, Huang AJ, Seal KH. Menopause symptoms and chronic pain in a national sample of midlife women veterans. Menopause. 2019;26(7):708–13. 10.1097/GME.0000000000001312. 30839364 10.1097/GME.0000000000001312

[22] Labrenz F, Merz CJ, Icenhour A. Connecting dots in disorders of gut-brain interaction: the interplay of stress and sex hormones in shaping visceral pain. Front Psychiatry. 2023;14:1204136. 10.3389/fpsyt.2023.1204136. 37275987 10.3389/fpsyt.2023.1204136 PMC10235543

[23] Sarnoff RP, Hreinsson JP, Sperber AD, Palsson OS, Bangdiwala SI, Chang L. Tu1029 effect of menopause and menses on gastrointestinal symptoms among individuals with DGBI in the global population. Gastroenterology. 2024;166(5):S1228-9. 10.1016/S0016-5085(24)03273-6.

[24] Cain KC, Jarrett ME, Burr RL, Rosen S, Hertig VL, Heitkemper MM. Gender differences in gastrointestinal, psychological, and somatic symptoms in irritable bowel syndrome. Dig Dis Sci. 2009;54(7):1542–9. 10.1007/s10620-008-0516-3. 18979200 10.1007/s10620-008-0516-3 PMC3569485

[25] Sheehan DV, Lecrubier Y, Sheehan KH, Amorim P, Janavs J, Weiller E, et al. The mini-international neuropsychiatric interview (M.I.N.I.): the development and validation of a structured diagnostic psychiatric interview for DSM-IV and ICD-10. J Clin Psychiatry. 1998;59(Suppl 20):22–33. 9881538

[26] Harlow SD, Gass M, Hall JE, Lobo R, Maki P, Rebar RW, et al. Executive summary of the stages of reproductive aging workshop +10: addressing the unfinished agenda of staging reproductive aging. Climacteric. 2012;15(2):105–14. 10.3109/13697137.2011.650656. 22338612 10.3109/13697137.2011.650656 PMC3580996

[27] Zigmond AS, Snaith RP. The hospital anxiety and depression scale. Acta Psychiatr Scand. 1983;67(6):361–70. 10.1111/j.1600-0447.1983.tb09716.x. 6880820 10.1111/j.1600-0447.1983.tb09716.x

[28] Spielberg CD, Gorsuch RL, Lushene R, Vagg PR, Jacobs GA. Manual for the state-trait anxiety inventory. Washington, DC: Consulting Psychologists Press; 1983.

[29] Cohen S, Kamarck T, Mermelstein R. A global measure of perceived stress. J Health Soc Behav. 1983;24(4):385–96. 6668417

[30] Kroenke K, Spitzer RL, Williams JB. The PHQ-15: validity of a new measure for evaluating the severity of somatic symptoms. Psychosom Med. 2002;64(2):258–66. 11914441 10.1097/00006842-200203000-00008

[31] Pennebaker JW. The psychology of physical symptoms. NewYork: Springer; 1982.

[32] Barch DM, Albaugh MD, Baskin-Sommers A, Bryant BE, Clark DB, Dick AS, et al. Demographic and mental health assessments in the adolescent brain and cognitive development study: updates and age-related trajectories. Dev Cogn Neurosci. 2021;52:101031. 10.1016/j.dcn.2021.101031. 34742018 10.1016/j.dcn.2021.101031 PMC8579129

[33] Glasser MF, Smith SM, Marcus DS, Andersson JL, Auerbach EJ, Behrens TE, et al. The human connectome project’s neuroimaging approach. Nat Neurosci. 2016;19(9):1175–87. 10.1038/nn.4361. 27571196 10.1038/nn.4361 PMC6172654

[34] Dale AM, Fischl B, Sereno MI. Cortical surface-based analysis. I. Segmentation and surface reconstruction. Neuroimage. 1999;9(2):179–94. 10.1006/nimg.1998.0395. 9931268 10.1006/nimg.1998.0395

[35] Fair DA, Miranda-Dominguez O, Snyder AZ, Perrone A, Earl EA, Van AN, et al. Correction of respiratory artifacts in MRI head motion estimates. Neuroimage. 2020;208:116400. 10.1016/j.neuroimage.2019.116400. 31778819 10.1016/j.neuroimage.2019.116400 PMC7307712

[36] Destrieux C, Fischl B, Dale A, Halgren E. Automatic parcellation of human cortical gyri and sulci using standard anatomical nomenclature. Neuroimage. 2010;53(1):1–15. 10.1016/j.neuroimage.2010.06.010. 20547229 10.1016/j.neuroimage.2010.06.010 PMC2937159

[37] Edlow BL, Takahashi E, Wu O, Benner T, Dai G, Bu L, et al. Neuroanatomic connectivity of the human ascending arousal system critical to consciousness and its disorders. J Neuropathol Exp Neurol. 2012;71(6):531–46. 10.1097/NEN.0b013e3182588293. 22592840 10.1097/NEN.0b013e3182588293 PMC3387430

[38] Xu X, Roman JM, Issaq HJ, Keefer LK, Veenstra TD, Ziegler RG. Quantitative measurement of endogenous estrogens and estrogen metabolites in human serum by liquid chromatography-tandem mass spectrometry. Anal Chem. 2007;79(20):7813–21. 10.1021/ac070494j. 17848096 10.1021/ac070494j

[39] Xu X, Veenstra TD, Fox SD, Roman JM, Issaq HJ, Falk R, et al. Measuring fifteen endogenous estrogens simultaneously in human urine by high-performance liquid chromatography-mass spectrometry. Anal Chem. 2005;77(20):6646–54. 10.1021/ac050697c. 16223252 10.1021/ac050697c

[40] Flores R, Shi J, Fuhrman B, Xu X, Veenstra TD, Gail MH, et al. Fecal microbial determinants of fecal and systemic estrogens and estrogen metabolites: a cross-sectional study. J Transl Med. 2012;10:253. 10.1186/1479-5876-10-253. 23259758 10.1186/1479-5876-10-253 PMC3552825

[41] Meriwether D, Sulaiman D, Volpe C, Dorfman A, Grijalva V, Dorreh N, et al. Apolipoprotein A-I mimetics mitigate intestinal inflammation in COX2-dependent inflammatory bowel disease model. J Clin Invest. 2019;129(9):3670–85. 10.1172/JCI123700. 31184596 10.1172/JCI123700 PMC6715371

[42] Smith SM, Nichols TE. Threshold-free cluster enhancement: addressing problems of smoothing, threshold dependence and localisation in cluster inference. Neuroimage. 2009;44(1):83–98. 10.1016/j.neuroimage.2008.03.061. 18501637 10.1016/j.neuroimage.2008.03.061

[43] Winkler AM, Ridgway GR, Webster MA, Smith SM, Nichols TE. Permutation inference for the general linear model. Neuroimage. 2014;92(100):381–97. 10.1016/j.neuroimage.2014.01.060. 24530839 10.1016/j.neuroimage.2014.01.060 PMC4010955

[44] Winkler AM, Webster MA, Brooks JC, Tracey I, Smith SM, Nichols TE. Non-parametric combination and related permutation tests for neuroimaging. Hum Brain Mapp. 2016;37(4):1486–511. 10.1002/hbm.23115. 26848101 10.1002/hbm.23115 PMC4783210

[45] McIntosh AR, Lobaugh NJ. Partial least squares analysis of neuroimaging data: applications and advances. Neuroimage. 2004;23(Suppl 1):S250–63. 10.1016/j.neuroimage.2004.07.020. 15501095 10.1016/j.neuroimage.2004.07.020

[46] Menon V. Large-scale brain networks and psychopathology: a unifying triple network model. Trends Cogn Sci. 2011;15(10):483–506. 10.1016/j.tics.2011.08.003. 21908230 10.1016/j.tics.2011.08.003

[47] Bari A, Xu S, Pignatelli M, Takeuchi D, Feng J, Li Y, Tonegawa S. Differential attentional control mechanisms by two distinct noradrenergic coeruleo-frontal cortical pathways. Proc Natl Acad Sci USA. 2020;117(46):29080–9. 10.1073/pnas.2015635117. 33139568 10.1073/pnas.2015635117 PMC7682591

[48] Grueschow M, Kleim B, Ruff CC. Functional coupling of the locus coeruleus is linked to successful cognitive control. Brain Sci. 2022;12:3. 10.3390/brainsci12030305. 10.3390/brainsci12030305 PMC8946131 35326262

[49] Mather M, Clewett D, Sakaki M, Harley CW. Norepinephrine ignites local hotspots of neuronal excitation: how arousal amplifies selectivity in perception and memory. Behav Brain Sci. 2016;39:e200. 10.1017/S0140525X15000667. 26126507 10.1017/S0140525X15000667 PMC5830137

[50] Torromino G, Maggi A, De Leonibus E. Estrogen-dependent hippocampal wiring as a risk factor for age-related dementia in women. Prog Neurobiol. 2021;197:101895. 10.1016/j.pneurobio.2020.101895. 32781107 10.1016/j.pneurobio.2020.101895

[51] Todd AR, Forstmann M, Burgmer P, Brooks AW, Galinsky AD. Anxious and egocentric: how specific emotions influence perspective taking. J Exp Psychol Gen. 2015;144(2):374–91. 10.1037/xge0000048. 25602753 10.1037/xge0000048

[52] Vereb D, Mijalkov M, Canal-Garcia A, Chang YW, Gomez-Ruis E, Gerboles BZ, et al. Age-related differences in the functional topography of the locus coeruleus: implications for cognitive and affective functions. medRxiv. 2023. 10.1101/2023.02.25.23286442. 37650882 10.7554/eLife.87188 PMC10471162

[53] Sun J, Ma J, Gao L, Wang J, Zhang D, Chen L, et al. Disruption of locus coeruleus-related functional networks in Parkinson’s disease. NPJ Parkinsons Dis. 2023;9(1):81. 10.1038/s41531-023-00532-x. 37253752 10.1038/s41531-023-00532-x PMC10229645

[54] Jacobs HI, Wiese S, van de Ven V, Gronenschild EH, Verhey FR, Matthews PM. Relevance of parahippocampal-locus coeruleus connectivity to memory in early dementia. Neurobiol Aging. 2015;36(2):618–26. 10.1016/j.neurobiolaging.2014.10.041. 25433457 10.1016/j.neurobiolaging.2014.10.041

[55] Paden CM, McEwen BS, Fishman J, Snyder L, DeGroff V. Competition by estrogens for catecholamine receptor binding in vitro. J Neurochem. 1982;39(2):512–20. 10.1111/j.1471-4159.1982.tb03974.x. 7086432 10.1111/j.1471-4159.1982.tb03974.x

[56] Coulombe MA, Erpelding N, Kucyi A, Davis KD. Intrinsic functional connectivity of periaqueductal gray subregions in humans. Hum Brain Mapp. 2016;37(4):1514–30. 10.1002/hbm.23117. 26821847 10.1002/hbm.23117 PMC6867375

[57] Biswal B, Yetkin FZ, Haughton VM, Hyde JS. Functional connectivity in the motor cortex of resting human brain using echo-planar MRI. Magn Reson Med. 1995;34(4):537–41. 10.1002/mrm.1910340409. 8524021 10.1002/mrm.1910340409

[58] Jones LM, Fontanini A, Sadacca BF, Miller P, Katz DB. Natural stimuli evoke dynamic sequences of states in sensory cortical ensembles. Proc Natl Acad Sci U S A. 2007;104(47):18772–7. 10.1073/pnas.0705546104. 18000059 10.1073/pnas.0705546104 PMC2141852

[59] Bosak N, Branco P, Kuperman P, Buxbaum C, Cohen RM, Fadel S, et al. Brain connectivity predicts chronic pain in acute mild traumatic brain injury. Ann Neurol. 2022;92(5):819–33. 10.1002/ana.26463. 36082761 10.1002/ana.26463 PMC9826527

[60] Soldatelli M, Franco AO, Picon F, Duarte JA, Scherer R, Bandeira J, et al. Primary somatosensory cortex and periaqueductal gray functional connectivity as a marker of the dysfunction of the descending pain modulatory system in fibromyalgia. Korean J Pain. 2023;36(1):113–27. 10.3344/kjp.22225. 36581601 10.3344/kjp.22225 PMC9812696

[61] Fillingim RB, Ohrbach R, Greenspan JD, Sanders AE, Rathnayaka N, Maixner W, Slade GD. Associations of psychologic factors with multiple chronic overlapping pain conditions. J Oral Facial Pain Headache. 2020;34(Suppl):s85–100. 10.11607/ofph.2584. 32975543 10.11607/ofph.2584 PMC10165716

[62] Fillingim RB, Ohrbach R, Greenspan JD, Knott C, Diatchenko L, Dubner R, et al. Psychological factors associated with development of TMD: the OPPERA prospective cohort study. J Pain. 2013;14(12 Suppl):T75-90. 10.1016/j.jpain.2013.06.009. 24275225 10.1016/j.jpain.2013.06.009 PMC3855656

[63] Sperber AD, Bangdiwala SI, Drossman DA, Ghoshal UC, Simren M, Tack J, et al. Worldwide prevalence and burden of functional gastrointestinal disorders, results of rome foundation global study. Gastroenterology. 2021;160(1):99–114. 10.1053/j.gastro.2020.04.014. 32294476 10.1053/j.gastro.2020.04.014

[64] Harrison R, Gandhi W, van Reekum CM, Salomons TV. Conditioned pain modulation is associated with heightened connectivity between the periaqueductal grey and cortical regions. Pain Rep. 2022;7(3):e999. 10.1097/PR9.0000000000000999. 35558091 10.1097/PR9.0000000000000999 PMC9084428

[65] Ziegler K, Folkard R, Gonzalez AJ, Burghardt J, Antharvedi-Goda S, Martin-Cortecero J, et al. Primary somatosensory cortex bidirectionally modulates sensory gain and nociceptive behavior in a layer-specific manner. Nat Commun. 2023;14(1):2999. 10.1038/s41467-023-38798-7. 37225702 10.1038/s41467-023-38798-7 PMC10209111

[66] Xie Z, Feng J, Cai T, McCarthy R, Eschbach MD 2nd, Wang Y, et al. Estrogen metabolites increase nociceptor hyperactivity in a mouse model of uterine pain. JCI Insight. 2022;7:10. 10.1172/jci.insight.149107. 10.1172/jci.insight.149107 PMC9220826 35420999

[67] Peng HY, Chang HM, Lee SD, Huang PC, Chen GD, Lai CH, et al. TRPV1 mediates the uterine capsaicin-induced NMDA NR2B-dependent cross-organ reflex sensitization in anesthetized rats. Am J Physiol Renal Physiol. 2008;295(5):F1324–35. 10.1152/ajprenal.00126.2008. 18632800 10.1152/ajprenal.00126.2008

[68] Ford AC, Vanner S, Kashyap PC, Nasser Y. Chronic visceral pain: new peripheral mechanistic insights and resulting treatments. Gastroenterology. 2024;166(6):976–94. 10.1053/j.gastro.2024.01.045. 38325759 10.1053/j.gastro.2024.01.045 PMC11102851

[69] Balemans D, Boeckxstaens GE, Talavera K, Wouters MM. Transient receptor potential ion channel function in sensory transduction and cellular signaling cascades underlying visceral hypersensitivity. Am J Physiol Gastrointest Liver Physiol. 2017;312(6):G635–48. 10.1152/ajpgi.00401.2016. 28385695 10.1152/ajpgi.00401.2016

[70] van Wanrooij SJ, Wouters MM, Van Oudenhove L, Vanbrabant W, Mondelaers S, Kollmann P, et al. Sensitivity testing in irritable bowel syndrome with rectal capsaicin stimulations: role of TRPV1 upregulation and sensitization in visceral hypersensitivity? Am J Gastroenterol. 2014;109(1):99–109. 10.1038/ajg.2013.371. 24189713 10.1038/ajg.2013.371

[71] Terzian AL, Aguiar DC, Guimaraes FS, Moreira FA. Modulation of anxiety-like behaviour by transient receptor potential vanilloid type 1 (TRPV1) channels located in the dorsolateral periaqueductal gray. Eur Neuropsychopharmacol. 2009;19(3):188–95. 10.1016/j.euroneuro.2008.11.004. 19064314 10.1016/j.euroneuro.2008.11.004

[72] Madasu MK, Okine BN, Olango WM, Rea K, Lenihan R, Roche M, Finn DP. Genotype-dependent responsivity to inflammatory pain: a role for TRPV1 in the periaqueductal grey. Pharmacol Res. 2016;113:44–54. 10.1016/j.phrs.2016.08.011. 27520401 10.1016/j.phrs.2016.08.011

[73] Greicius MD, Krasnow B, Reiss AL, Menon V. Functional connectivity in the resting brain: a network analysis of the default mode hypothesis. Proc Natl Acad Sci U S A. 2003;100(1):253–8. 10.1073/pnas.0135058100. 12506194 10.1073/pnas.0135058100 PMC140943

[74] Buckner RL, Andrews-Hanna JR, Schacter DL. The brain’s default network: anatomy, function, and relevance to disease. Ann N Y Acad Sci. 2008;1124:1–38. 10.1196/annals.1440.011. 18400922 10.1196/annals.1440.011

[75] Wen Z, Pace-Schott EF, Lazar SW, Rosen J, Ahs F, Phelps EA, et al. Distributed neural representations of conditioned threat in the human brain. Nat Commun. 2024;15(1):2231. 10.1038/s41467-024-46508-0. 38472184 10.1038/s41467-024-46508-0 PMC10933283

[76] Webb EK, Huggins AA, Belleau EL, Taubitz LE, Hanson JL, deRoon-Cassini TA, Larson CL. Acute posttrauma resting-state functional connectivity of periaqueductal gray prospectively predicts posttraumatic stress disorder symptoms. Biol Psychiatry Cogn Neurosci Neuroimaging. 2020;5(9):891–900. 10.1016/j.bpsc.2020.03.004. 32389746 10.1016/j.bpsc.2020.03.004 PMC7483700

[77] Kucyi A, Moayedi M, Weissman-Fogel I, Goldberg MB, Freeman BV, Tenenbaum HC, Davis KD. Enhanced medial prefrontal-default mode network functional connectivity in chronic pain and its association with pain rumination. J Neurosci. 2014;34(11):3969–75. 10.1523/JNEUROSCI.5055-13.2014. 24623774 10.1523/JNEUROSCI.5055-13.2014 PMC6705280

[78] Jenness JL, Jager-Hyman S, Heleniak C, Beck AT, Sheridan MA, McLaughlin KA. Catastrophizing, rumination, and reappraisal prospectively predict adolescent PTSD symptom onset following a terrorist attack. Depress Anxiety. 2016;33(11):1039–47. 10.1002/da.22548. 27557454 10.1002/da.22548 PMC5325818

[79] Olff M. Sex and gender differences in post-traumatic stress disorder: an update. Eur J Psychotraumatol. 2017. 10.1080/20008198.2017.1351204.30128082

[80] Ditlevsen DN, Elklit A. The combined effect of gender and age on post traumatic stress disorder: do men and women show differences in the lifespan distribution of the disorder? Ann Gen Psychiatry. 2010;9:32. 10.1186/1744-859X-9-32. 20663164 10.1186/1744-859X-9-32 PMC2917414

[81] Appels R, Eversole K, Feuillet C, Keller B, et al. Shifting the limits in wheat research and breeding using a fully annotated reference genome. Science. 2018. 10.1126/science.aar7191. 30115783 10.1126/science.aar7191

