# Supplementary material for: Differential brainstem connectivity according to sex and menopausal status in healthy male and female individuals
Source: Biol Sex Differ. 2025 Apr 18;16:25. doi: 10.1186/s13293-025-00709-4 (PMC12007138; doi:10.1186/s13293-025-00709-4)
Supplement: Supplementary file 1 — Supplementary Material 1. Mean estrogen levels, with standard errors, in males, premenopausal females, and postmenopausal females. Plasma data is provided in units of ng/mL-plasma, while stool data is provided in units of ng/5 mL-stool. E1, estrone; E2, estradiol; E3, estriol: 2OHE1, 2-hydroxyestrone; 2MeOE1, 2-methoxyestrone; 2OHE2, 2-hydroxyestradiol; 2MeOE2, 2-methoxyestradiol; 3MeOE1, 2-hydroxyestrone-3-methyl ether; 4OHE1, 4-hydroxyestrone; 4MeOE1, 4-methoxyestrone; 4MeOE2, 4-methoxyestradiol; 16aOHE1, 16α-hydroxyestrone; 17epiE3, 17-epiestriol; 16ketoE2, 16-ketoestradiol; 16epiE3, 16-epiestriol; Total, summation of all free or totalestrogens and estrogen metabolites, dependent on category. [file 13293_2025_709_MOESM1_ESM.pdf]

Additional File 1 (pdf): Mean estrogen levels, with standard errors, in males, premenopausal females, and postmenopausal females. Plasma data is provided in units of ng/mL-plasma, while stool data is provided in units of ng/5mL-stool. E1, estrone; E2, estradiol; E3, estriol: 2OHE1, 2-hydroxyestrone; 2MeOE1, 2-methoxyestrone; 2OHE2, 2-hydroxyestradiol; 2MeOE2, 2-methoxyestradiol; 3MeOE1, 2-hydroxyestrone-3-methyl ether; 4OHE1, 4-hydroxyestrone; 4MeOE1, 4-methoxyestrone; 4MeOE2, 4-methoxyestradiol; 16aOHE1, 16α-hydroxyestrone; 17epiE3, 17-epiestriol; 16ketoE2, 16-ketoestradiol; 16epiE3, 16-epiestriol; Total, summation of all free or total (free + conjugated) estrogens and estrogen metabolites, dependent on category

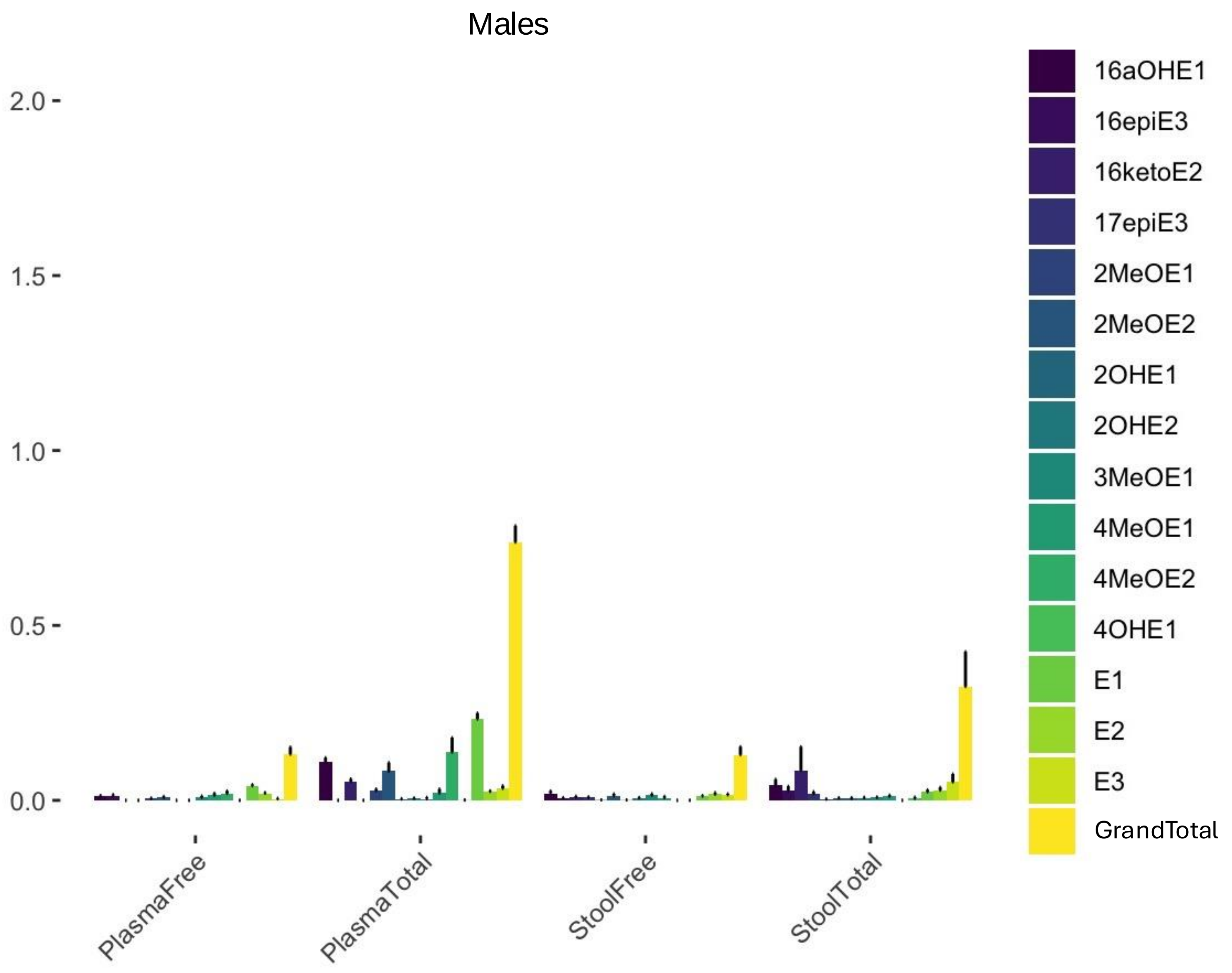

Males
2.0
1.5
1.0
0.5
0.0
PlasmaFree
PlasmaTotal
StoolFree
StoolTotal
16aOHE1
16epiE3
16ketoE2
17epiE3
2MeOE1
2MeOE2
2OHE1
2OHE2
3MeOE1
4MeOE1
4MeOE2
4OHE1
E1
E2
E3
GrandTotal

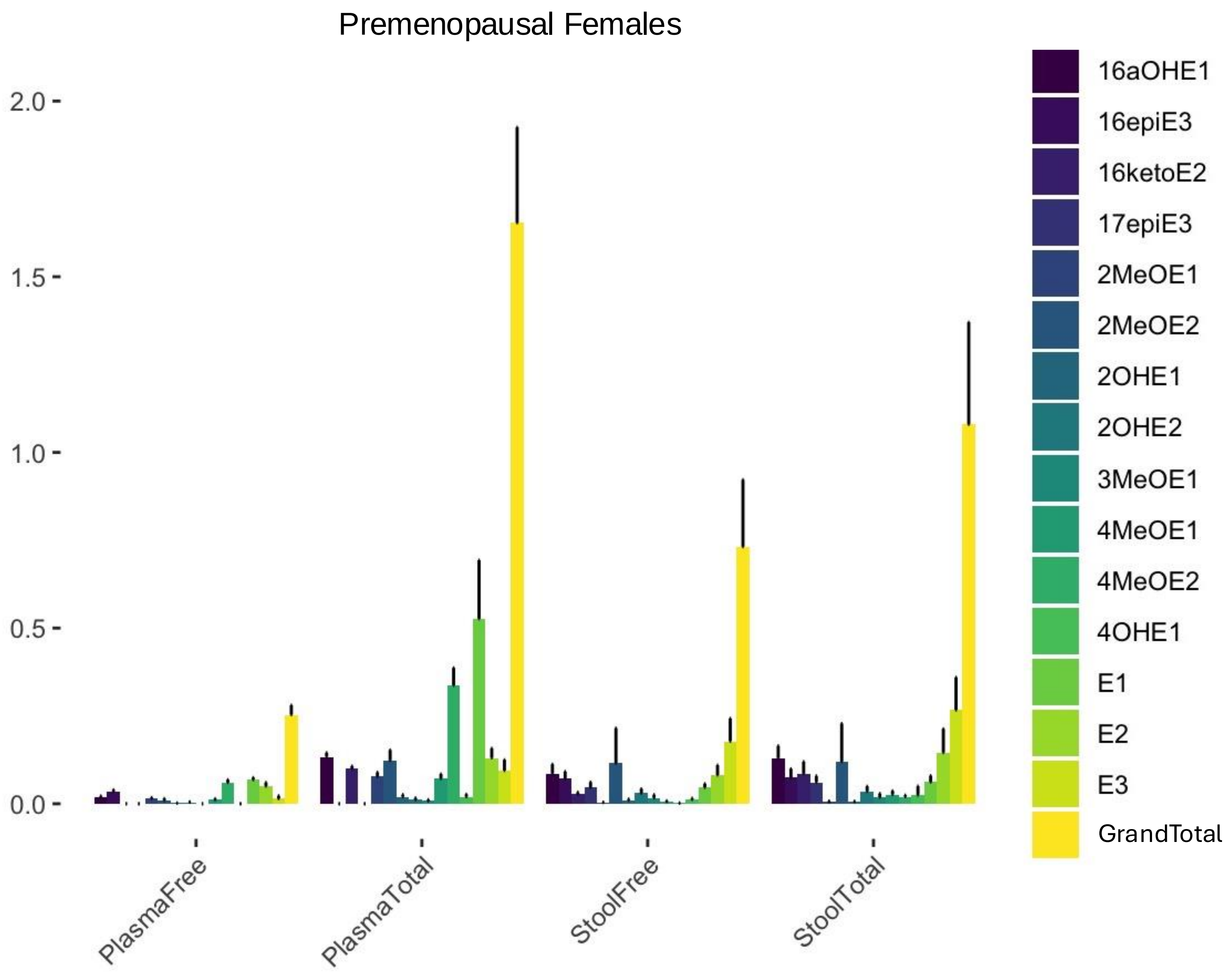

Premenopausal Females
2.0
1.5
1.0
0.5
0.0
PlasmaFree
PlasmaTotal
StoolFree
StoolTotal
16aOHE1
16epiE3
16ketoE2
17epiE3
2MeOE1
2MeOE2
2OHE1
2OHE2
3MeOE1
4MeOE1
4MeOE2
4OHE1
E1
E2
E3
GrandTotal

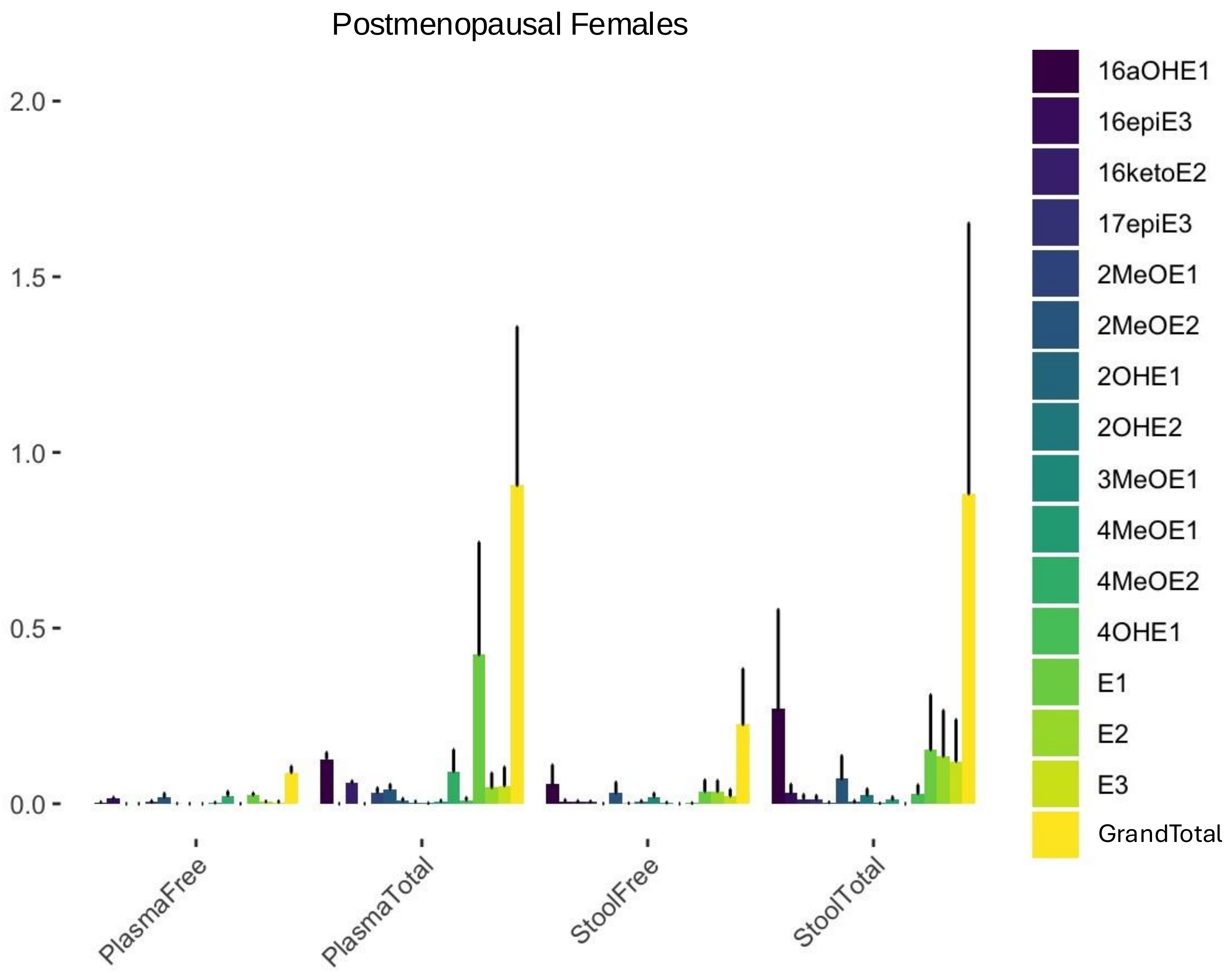

Postmenopausal Females
2.0
1.5
1.0
0.5
0.0
PlasmaFree
PlasmaTotal
StoolFree
StoolTotal
16aOHE1
16epiE3
16ketoE2
17epiE3
2MeOE1
2MeOE2
2OHE1
2OHE2
3MeOE1
4MeOE1
4MeOE2
4OHE1
E1
E2
E3
GrandTotal
